# Supplementary figures and images for: Factors influencing subclinical atherosclerosis in patients with biopsy-proven nonalcoholic fatty liver disease
Source: PLoS One. 2019 Nov 13;14(11):e0224184. doi: 10.1371/journal.pone.0224184 (PMC6853607; doi:10.1371/journal.pone.0224184)

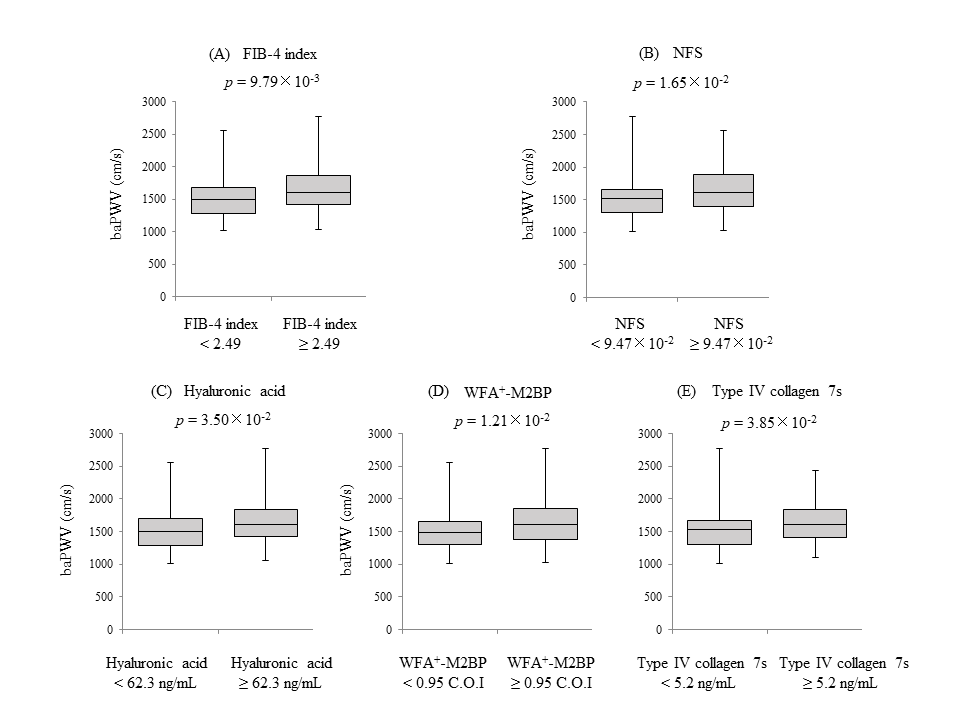

Supplement: S1 Fig — Box and whisker plots of baPWV values according to each fibrosis marker and score such as FIB-4 index (A), NFS (B), hyaluronic acid (C), WFA+-M2BP (D), and type IV collagen 7s (E). baPWV, brachial-ankle pulse wave velocity; FIB-4, fibrosis-4; NFS, NAFLD (nonalcoholic fatty liver disease) fibrosis score; WFA+-M2BP, Wisteria floribunda agglutinin positive Mac-2-binding protein. (TIF) [file pone.0224184.s001.tif]
